# Brain volumetry and spinal cord imaging in patients with AQP4-IgG+ NMOSD—a systematic review and meta-analysis

**DOI:** 10.1177/17562864251394843

**Published:** 2025-12-06

**Authors:** Josephine Heine, Claudia Chien

**Affiliations:** Department of Psychiatry and Neurosciences, Charité—Universitätsmedizin Berlin, Corporate Member of Freie Universität Berlin and Humboldt-Universität zu Berlin, Charitéplatz 1, Berlin 10117, Germany; Experimental and Clinical Research Center, Charité—Universitätsmedizin Berlin, Corporate Member of Freie Universität Berlin and Humboldt-Universität zu Berlin, Berlin, Germany; Department of Psychiatry and Neurosciences, Charité—Universitätsmedizin Berlin, Corporate Member of Freie Universität Berlin and Humboldt-Universität zu Berlin, Berlin, Germany; Experimental and Clinical Research Center, Charité—Universitätsmedizin Berlin, Corporate Member of Freie Universität Berlin and Humboldt-Universität zu Berlin, Berlin, Germany; Neuroscience Clinical Research Center, Charité—Universitätsmedizin Berlin, Corporate Member of Freie Universität Berlin and Humboldt-Universität zu Berlin, Berlin, Germany

**Keywords:** aquaporin-4, deep gray matter, lesion volume, magnetic resonance imaging, MUCCA, neuromyelitis optica spectrum disorder, segmentation, thalamus, volumetry, voxel-based morphometry

## Abstract

**Background:**

Magnetic resonance imaging (MRI) is often used to evaluate disease-related brain changes in patients with aquaporin-4-IgG seropositive neuromyelitis optica spectrum disorder (AQP4-IgG+ NMOSD).

**Objectives:**

To use a meta-analysis for assessment of quantitative volumetric brain and spinal cord changes in patients with AQP4-IgG+ NMOSD and healthy participants.

**Design:**

We analyzed volume estimates of the brain, gray matter, white matter, thalamus, T2/FLAIR-brain lesions, as well as mean upper cervical cord area (MUCCA). Inclusion criteria included patients with AQP4-IgG+ NMOSD, MRI-based segmentation data, and matched healthy participants. Data from NMOSD patients with mixed/unknown serostatus or significant comorbidities were excluded.

**Data sources:**

We searched MEDLINE through Pubmed for peer-reviewed articles published between 05/2006 (revised NMOSD diagnostic criteria) and 01/2025.

**Methods:**

Standardized mean differences and pooled effect sizes (Hedges’ *g*) were determined with random-effects models, adjusting for duplicate reporting, outliers, and small study effects. Metaregressions were used to determine clinical associations.

**Results:**

Evidence of pooled data showed that whole brain volume (*g* = −0.61, 95% confidence interval (CI): −0.91 to −0.32, *p* < 0.001, *N*_pat/con_ = 385/325, *k* = 11) and gray matter volume (*g* = −0.40, 95% CI: −0.72 to −0.09, *p* = 0.018, *N*_pat/con_ = 259/267, *k* = 9) were significantly different between patients and healthy participants. Heterogeneity was moderate (τ² = 0.08 and τ² = 0.09, respectively). Moreover, we found a large effect for reduced MUCCA (*g* = −0.99, 95% CI: −1.59 to −0.39, *p* = 0.007, *N*_pat/con_ = 189/162, *k* = 7) with moderate heterogeneity (τ² = 0.31). No conclusive evidence emerged for changes in thalamic or white matter volume. Bias analysis did not indicate that smaller studies affected effect sizes. A systematic review of voxel-based morphometry revealed that reduced gray matter volume was most likely in the bilateral thalamus (⩽69%) and occipital (44%), frontal (27%), and temporal cortices (27%).

**Conclusion:**

AQP4-IgG+ NMOSD patients have specific global and local central nervous system volume reductions, potentially induced by astrocytic damage and demyelination. Volumetric outcomes may therefore inform MRI-guided disease monitoring and endpoints in clinical studies.

**Trial registration:**

PROSPERO (CRD42024493121). This study follows the Preferred Reporting Items for Systematic Reviews and Meta-Analyses reporting guidelines.

## Plain language summary

A combined statistical review of brain and spinal cord damage measured by magnetic resonance imaging in people with neuromyelitis optica spectrum disorder

**Why was the study done?** Aquaporin-4-IgG seropositive neuromyelitis optica spectrum disorder (AQP4-IgG+ NMOSD) is a rare disease that mostly affects the brain and spinal cord. Brain scans taken with magnetic resonance imaging (MRI) are often used to detect damage in the central nervous system. To get a clearer picture of how AQP4-IgG+ NMOSD affects brain volumes and the spinal cord, this article was written by combining data from many previous studies, otherwise known as a “meta-analysis” study.

**What did the researchers do?** The research team looked at MRI studies of adults (18 years of age and older) with confirmed AQP4-IgG+ NMOSD and healthy people of similar age and sex. Measurements of total brain volume, gray and white matter, the thalamus, spinal cord area, and brain lesions were analyzed. The meta-analysis used statistical techniques to find overarching trends in the data.

**What did the researchers find?** People with AQP4-IgG+ NMOSD had moderately smaller total brain and gray matter volumes than healthy individuals. The strongest result was for spinal cord size: the upper cervical part of the spinal cord was significantly smaller in patients. Evidence for white matter and thalamus volume differences in patients was not shown to be a strong finding in most studies analyzed. There were no signs that small or low-quality studies had influenced the results of this meta-analysis. Gray matter loss was found most often in the thalamus and parts of the visual, frontal, and temporal brain regions.

**What do the findings mean?** Overall, the findings show that AQP4-IgG+ NMOSD causes loss of brain tissue in several regions of the brain. The size of the spinal cord is also affected in patients with this disease, especially in the upper cervical region. Measuring central nervous system regions from MRI can therefore help researchers to understand how people with AQP4-IgG+ NMOSD are affected over the years of this chronic disease. These findings from this article can also be useful to look at when testing effective treatments in clinical studies.

## Introduction

Neuromyelitis optica spectrum disorder (NMOSD) is a severe inflammatory disorder marked by relapsing inflammation of the optic nerve and spinal cord.^1,2^ Acute attacks can cause optic neuritis, transverse myelitis, or area postrema syndrome, leading to muscle weakness, sensory loss, paralysis, visual impairment, nausea, or fatigue. More than 80% of the patients with NMOSD test positive for immunoglobulin G autoantibodies against the astrocyte water channel aquaporin-4 (AQP4).^3^ Principally, damage occurs to astrocytes in AQP4-IgG+ NMOSD, but secondary loss of cortical neurons and oligodendrocytes has been described as well.^4–6^

Brain and spinal cord magnetic resonance imaging (MRI) plays a central role in the diagnosis and prognostication of NMOSD. Indeed, most of the core clinical characteristics necessary for a diagnosis of AQP4-IgG+ NMOSD rely on findings based on MRI.^2^ Here, longitudinally extensive transverse myelitis lesions of the spinal cord, as well as lesions in the optic nerve, area postrema, or medulla are common.^7^ Recent findings have also shown that brain lesions are more prevalent than previously assumed and affect the majority of patients.^8–10^

This pattern also underscores the use of MRI to distinguish between AQP4-IgG+ NMOSD and its mimics, such as multiple sclerosis (MS) and myelin oligodendrocyte glycoprotein antibody-associated disease (MOGAD), to provide targeted treatment.^11–13^ NMOSD, for instance, is not generally associated with disease-characteristic brain lesions, such as the Dawson’s fingers of the corpus callosum often seen in patients with MS.^11^ Moreover, a recent comprehensive meta-analysis found that patients with MS showed lower gray matter and thalamus volumes, together with higher T2 lesion volumes and T1 lesion counts, than patients with NMOSD.^14^ In contrast, atrophy in the occipital cortex was more prominent in AQP4-IgG+ NMOSD than in MS in a multicenter MRI study.^15^

Advanced MRI postprocessing techniques, such as the automated segmentation of brain regions and extraction of volume estimates, can be useful tools to refine the monitoring of disease activity, identify subclinical progression, and in developing prognostic markers.^12^ However to date, volumetric studies in NMOSD have yielded inconsistent results. Volume reductions may depend on serostatus, with studies frequently reporting heterogeneous samples that include patients with unknown serostatus, seronegative, or double-seropositive NMOSD. Not all patients who were seronegative for AQP4-IgG antibodies are subsequently tested for MOG antibodies. This may create an additional level of heterogeneity pertaining to serostatus. Moreover, some studies lack a negative control group of healthy participants matched for demographic characteristics. Comparisons with MOGAD and seronegative NMOSD groups are informative for clinical observations related to AQP4-IgG+ NMOSD, but quantitative MRI metrics in relation to the healthy population still lack conclusive evidence. In the same vein, collated evidence on comparable volumetric MRI metrics is necessary to facilitate the choice of relevant imaging endpoints in future clinical trials.

This review therefore aims to provide an overview of volumetric MRI in the distinct subgroup of patients with AQP4-seropositive NMOSD. Using data extracted from MRIs of patients with AQP4-IgG+ NMOSD and healthy age- and sex-matched participants, we perform meta-analyses for brain volumes, including whole brain, gray matter, white matter, and thalamus volume, as well as the mean upper cervical cord area (MUCCA). Finally, we review findings of studies that use voxel-based morphometry (VBM), a semi-automated MRI volume estimation method, to describe cortical and subcortical gray matter volume reductions in patients with AQP4-IgG+ NMOSD.

## Methods

### Registration and protocol

This study was prospectively registered with PROSPERO (https://www.crd.york.ac.uk/PROSPERO/view/CRD42024493121) and conducted according to the Preferred Reporting Items for Systematic Reviews and Meta-Analyses (PRISMA) guidelines.^16^ Differences between the registered protocol and the final version are the addition of Hedge’s *g* as an effect measure as part of the meta-analysis and additional risk of bias assessment using Egger’s regression for small study effects. Inclusion in the volumetric meta-analysis furthermore required an age-matched control group of healthy participants, except for brain lesion volumes. Lastly, demographic subgroup analysis was omitted and instead operationalized as a metaregression.

### Eligibility criteria

We included studies meeting the following inclusion criteria: (1) peer-reviewed journal articles reporting (2) original volumetric data from (3) in vivo MRI in (4) adult human patients (⩾18 years of age) with (5) a diagnosis of AQP4-IgG seropositive NMOSD and (6) without significant comorbidities. Diagnostic criteria included the Wingerchuk 2015 criteria for NMOSD with AQP4-IgG for articles published after July 2015.^2^ Articles published between May 2006 and July 2015 were included if patients met the Wingerchuk 2006 revised criteria for definite NMO with the third supportive criterion (“NMO-IgG seropositive status”).^17^

We excluded articles reporting data from Myelin Oligodendrocyte Glycoprotein-IgG (MOG) seropositive, AQP4/MOG-IgG double seropositive, or seronegative patients. Studies involving pediatric patients, postmortem imaging, animal studies, as well as patients with significant neurological comorbidities at the time of imaging (e.g., myasthenia gravis, glioblastoma, Wernicke’s encephalopathy, T-cell lymphoma, breast/bladder cancer, neurosyphilis) were also excluded. Reports in the form of conference abstracts, posters, or preprints were not considered.

### Search strategy

The MEDLINE database was searched through PubMed using the following query: (“NMO”[Title/Abstract] OR “NMOSD”[Title/Abstract] OR “neuromyelitis optica”[Title/Abstract]) AND (“imaging”[Title/Abstract] OR “neuroimaging”[Title/Abstract] OR “magnetic resonance imaging”[Title/Abstract] OR “positron emission tomography”[Title/Abstract] OR “optical coherence tomography”[Title/Abstract] OR “OCT”[Title/Abstract] OR “MRI”[Title/Abstract] OR “PET”[Title/Abstract]) AND (“2006/01/01”[Date–Publication] : “2024/09/01”[Date–Publication]) AND (English[Language]) NOT (Preprint[Publication Type]). Searches were restricted to articles published in English between May 22, 2006 (publication of the revised diagnostic criteria) and September 01, 2024, and re-run to identify articles published between the end of the search period and the final analysis on January 31, 2025. Additionally, we identified potential studies through searching key journals (see Supplemental Methods) and reference lists of eligible studies and review articles.

### Study selection and data cleaning

Reports were screened and data were extracted by one reviewer (J.H.). The second reviewer independently selected studies at random and conducted an internal quality control (C.C.). Ambiguous cases were discussed by both reviewers until reaching consensus. Data extraction was performed in Microsoft Excel (version 2504).

Clinical, volumetric, scanner, and publication meta-data were extracted from articles and available Supplemental Material. For longitudinal studies, we included the baseline time point. If subgroups of patients were reported (e.g., with and without cognitive impairment), each group was entered separately provided they fulfilled all inclusion criteria. In cases in which only a part of the study sample was AQP4-seropositive, we contacted the authors of the study to obtain the data for the AQP4-seropositive patients only. Missing data were requested from the study authors or otherwise coded as “NA” (“not available”).

### Data items

Three main criteria were used to select MRI measures of interest for the meta-analysis. (I) Relevance: the region of the central nervous system (CNS) had to be implicated in the disease course of NMOSD, for example, through being related to NMOSD symptoms (i.e., spinal cord) or reflecting possible disease activity (i.e., lesion volume). (II) Availability: the MRI measure needed to be frequently reported across studies (*k* ⩾ 5 studies suitable for inclusion). (III) Comparability: the methodological approaches had to be sufficiently robust, that is, through applying well validated semi-automated segmentation algorithms as in FSL SIENAX,^18^ FSL FIRST,^19^ and Freesurfer,^20^ as well as using similar MRI acquisition parameters (such as voxel size or MRI sequence).

Based on these criteria, we included the following variables of interest: whole brain volume (mm^3^), white and gray matter volume (mm^3^), thalamus volume (mm^3^), and MUCCA (mm^2^). In patients, brain lesion volume (ml) was included to examine associations with clinical variables. Clinical features, including sex, age, disease duration, the Expanded Disability Status Scale (EDSS), the number of attacks, and history of optic neuritis/myelitis/brainstem syndrome/area postrema syndrome, were also included in the data extraction. Types of therapies and treatments were classified into: treatment of acute attacks (glucocorticoids, methotrexate, methylprednisolone, plasma exchange, prednisolone), attack-preventing therapy (azathioprine, eculizumab, inebilizumab, mycophenolate mofetil, rituximab, satralizumab, tocilizumab), and other treatment options that are considered unconventional (anti-CD20 therapy, beta interferon, cyclophosphamide, glatiramer acetate, intravenous immunoglobulins, mitoxantrone).^21–23^

### Data synthesis

To prepare the data for the combined analysis, we performed the following data transformations.

#### Pooling of continuous outcomes

Data reported as median and range were transformed to mean and standard deviation based on the sample size-based approach established by Hozo et al.^24^ Data reported as median and interquartile range were transformed to mean and standard deviation using the quantile estimation method by McGrath et al.^25^

#### Volumetric units

Brain lesion volumes (if reported in cm^3^ or mm^3^) were transformed to milliliter (ml). Brain volumes (if reported in cm^3^ or ml) were transformed to cubic millimeter (mm^3^). Mean volumes were used for volume estimates of bilateral brain structures. For instance, when left and right volumes were reported separately, the average volume of the two sides was calculated. When studies reported the sum of left and right volume, this volume was divided by 2.

#### Time units

Time reported in months were transformed to years. When the annualized relapse rate (ARR) was reported, the number of relapses was estimated by multiplying the ARR by the disease duration in years.

### Meta-analysis, effect measures, and certainty assessment

Statistical analyses were performed using the *meta*,^26^ *metafor*,^27^ and *dmetar*^28^ packages in R 4.5.0. (R Core Team, 2025, https://www.r-project.org/).^29^ Volumetric outcomes with a minimum of *k* ⩾ 5 available studies meeting the inclusion criteria were included. Bias-corrected standardized mean differences (SMD) are reported as Hedges’ *g* using exact formulae. To pool effect sizes, we used a random-effects model to account for the expected between-study heterogeneity frequently seen in rare diseases. The heterogeneity variance (τ²) was determined using a restricted maximum likelihood estimator,^30^ including confidence intervals (CIs) based on Knapp–Hartung adjustments.^31^ For reference, we additionally quantify between-study heterogeneity using *I*^2^ and based on Cochran’s *Q*.^32^

To examine associations between MRI and clinical measures across studies, we performed a multiple meta-regression with *N* = 1000 permutations. After inspecting all variables for multicollinearity using intercorrelation matrices, mixed-effects regression models with sex, age, disease duration, disease-related disability (EDSS), frequency of immunotherapy treatments, and mean brain lesion volume were fitted as predictors. Potential confounding effects due to differences in data acquisition and segmentation approaches were tested using the non-parametric Kruskal–Wallis test.

### Risk of bias assessment

#### Multiple publications

Clinical centers often publish multiple studies based on the same patient cohort, particularly in rare diseases. This may lead to overlaps in study samples. To avoid potential sample duplication, each MRI outcome was screened for potential duplicates. Studies with high risk of sample overlaps were identified based on the reported information on clinical centers, patient recruitment, as well as author lists and affiliations. In case of a likely overlap, one study per center was selected for each MRI outcome based on largest sample size, availability of a matched healthy control group, and amount of clinical information provided.

#### Publication bias

According to the “file drawer problem,” studies yielding significant effects in the expected direction may be more likely to be published than studies with null or small effects. To counter this bias, we included studies meeting the inclusion criteria regardless of size and direction of the effect. Moreover, we performed an automated outlier detection using the *dmetar* R package.^28^ Here, outliers were identified if a study’s 95% CI lay outside of the 95% CI of the pooled effect. The analysis was then subsequently recalculated without the outlier.

#### Citation bias

Studies were assessed based on the inclusion criteria, irrespective of the number of citations they received and whether they reported significant group effects or null effects.

#### Small study effects

Given the rarity of the disease, it is not uncommon that studies report relatively small sample sizes. Since small samples sizes can potentially affect the standard errors, we evaluated small-study effects using funnel plots and, in addition, calculated Egger’s regression test.^33,34^ In cases where a significant Egger’s test suggested a potential publication bias, the pooled effect size estimates (*g*) were corrected using the Duval and Tweedie trim and fill method.^35^ Adjusted effect size estimates are reported as *g*_adj_.

#### Effects of segmentation approaches

Differences in data acquisition or segmentation software may impact the obtained volumetric measures. To control for this bias, we therefore evaluated the effects of scanner type, field strength, voxel size, segmentation software, and whether lesion filling was performed.

#### Technological advances

The “year of study” was used as a proxy for improvements in measurement accuracy over this period, running meta-regressions on effect size versus publication year to estimate the impact of cumulative methodological advances on volumetric methods.

### Systematic review of whole brain volumetry

Besides analyzing volume estimates extracted from regions of interest, we systematically reviewed studies reporting VBM. This automated technique allows to assess localized cortical and subcortical gray matter changes on a whole-brain basis. Selection criteria were a diagnosis of NMOSD, an exclusively AQP4-seropositive sample, and the availability of an age- and sex-matched healthy control group. Sample sizes, locations, and frequencies of local volume reductions were recorded. Frequencies are reported in percent and represent the proportion of affected versus nonaffected patients across studies, that is, the likelihood of a brain region showing a group difference when scaled for sample size. Plots were created using *ggseg*^15^ and SurfIce (https://www.nitrc.org/projects/surfice/).

## Results

### Study selection

**Figure 1.**
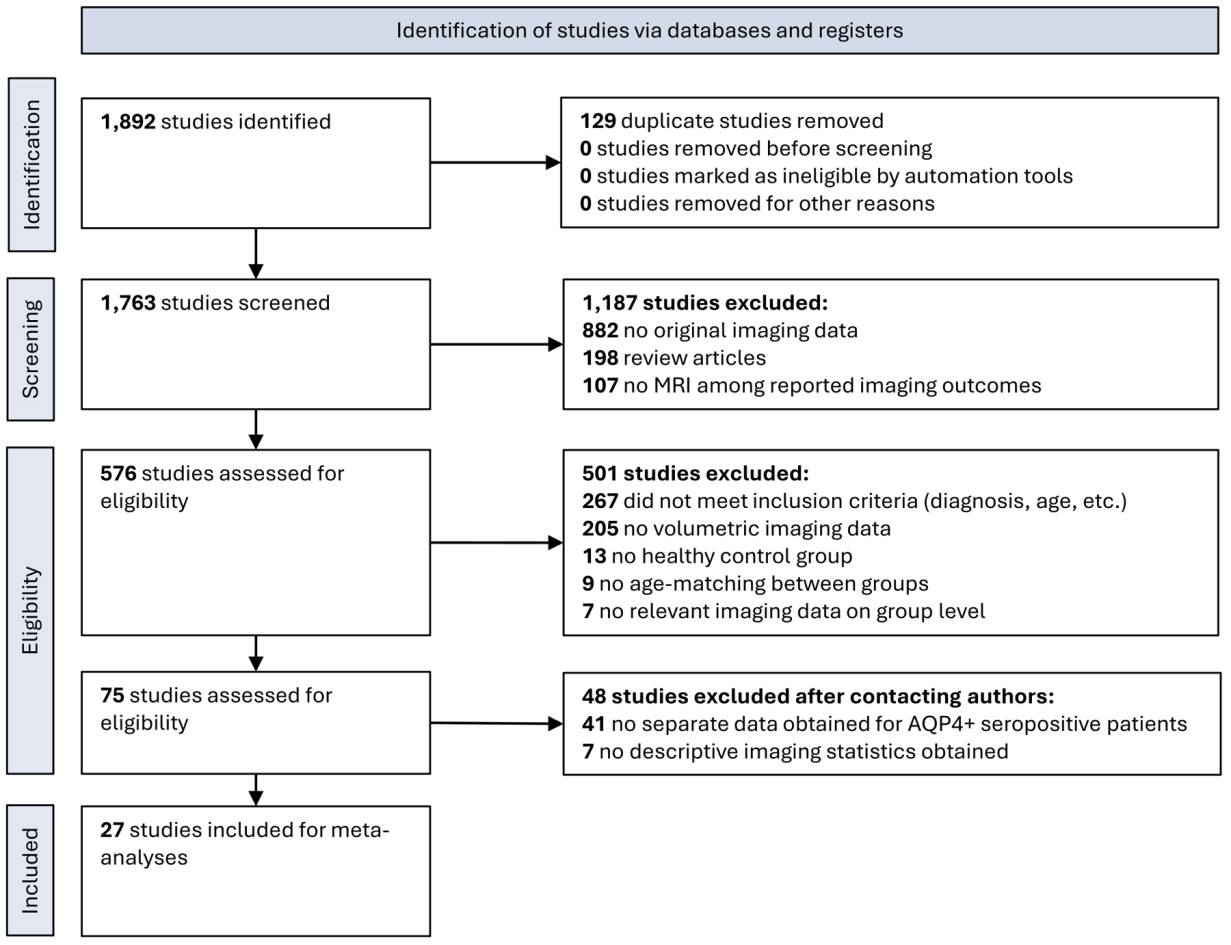
PRISMA flow diagram delineating the screening and inclusion process. PRISMA, Preferred Reporting Items for Systematic Reviews and Meta-Analyses.

A systematic search resulted in 1892 studies. Removal of duplicate studies and screening for inclusion criteria yielded 75 potentially eligible studies. After obtaining additional data from authors, a total of 27 studies from 16 countries were included (see PRISMA flow diagram in Figure 1). Clinical centers were located in Brazil (one center), Canada (one center), China (four centers), Cuba (one center), Czech Republic (one center), France (one center), Germany (one center), Italy (two centers), Japan (two centers), Poland (one center), Serbia (one center), South Korea (two centers), the United Kingdom (four centers), and the US (one center). An overview of the study characteristics and details on the screening for multiple reporting are provided in Table 1.

**Table 1.** Study inclusion.

No.	Country	Center	Reference	*N*		Volumes included						Multiple reports (excluded)
				Patients	Controls	Whole brain	T2 lesion	Gray matter	White matter	Thalamus	MUCCA	Chai et al.^36^ Cortese et al.^15^
1	Brazil	Hospital das Clínicas, Universidade de São Paulo, São Paulo	Boaventura et al.^37^	59	–	–	X	–	–	–	–	Silveira et al.^38^
2	Canada	University of British Columbia, Vancouver	Tsai et al.^39^	11	21	X	X	X	X	X	–	Manogaran et al.^40^
3	China	Tiantan Hospital, Capital Medical University, Beijing	Sun et al.^41^	99	48	X	X	X	X	–	–	Duan et al.,^42^ Gao et al.,^43^ Zhuo et al.^44^
4		Xuanwu Hospital, Capital Medical University, Beijing	Liu et al.^45^	25	20	X	–	–	–	X	X	–
5		First Affiliated Hospital of Nanchang University, Nanchang	Wang et al.^46^	30	45	X	X	X	X	–	–	–
6		Tianjin Medical University General Hospital, Tianjin	Sun et al.^47^	22	30	–	X	X	–	–	–	Li et al.^48^
7	Cuba	Center for Neurological Restoration, Havana	Sánchez-Catasús et al.^49^	9	15	–	–	X	X	–	–	–
8	Czech Republic	General University Hospital, Charles University, Prague	Pudlac et al.^50^	20	20	–	–	–	–	X	–	–
9	France	University Hospital Strasbourg, Strasbourg	Chanson et al.^51^	16	30	X	–	X	X	–	–	–
10			Lersy et al.^52^	9	9	–	–	–	–	–	X	–
11	Germany	Charité University Clinic, Berlin	Chien et al.^53^	40	31	X	X	–	–	–	X	Asseyer et al.,^54,55^ Chien et al.,^56,57^ Heine et al.,^58^ Komnenić et al.^59^
12			Finke et al.^60^	36	36	–	–	–	–	X	–	Chien et al.,^53^ Papadopoulou et al.^61^
13	Italy	IRCCS San Raffaele Scientific Institute, Milan	Savoldi et al.^62^	25	30	–	–	X	X	X	–	Cacciaguerra et al.^63–65^
14	Italy,^a^ Serbia^b^	^a^IRCCS San Raffaele Scientific Institute, Milan; ^b^University of Belgrade, Belgrade	Cacciaguerra et al.^66^	52	28	X	–	–	–	–	X	Cacciaguerra et al.,^63–65^ Savoldi et al.^62^
15			Cacciaguerra et al.^67^	85	–	–	X	–	–	–	–	Cacciaguerra et al.,^63–66,68^ Rocca et al.,^69^ Savoldi et al.^62^
16	Japan	Chiba University Hospital, Chiba	Masuda et al.^70^	29	29	X	X	X	X	–	–	Masuda et al.^71,72^
17		Juntendo University Graduate School of Medicine, Tokyo	Andica et al.^73^	18	19	X	–	X	–	X	–	–
18			Kato et al.^74^	18	–	–	X	–	–	–	–	–
19	Poland	Warsaw Medical University and Wolski Hospital, Warsaw	Jakuszyk et al.^75^	20	–	–	X	–	–	–	–	–
20	South Korea	St Mary’s Hospital, The Catholic University of Korea, Seoul	Kim et al.^76^	15	–	–	X	–	–	–	–	–
21		Seoul National University Hospital, Seoul	Kim et al.^77^	21	–	–	X	–	–	–	–	–
22	UK^c,d^	^c^National Hospital for Neurology and Neurosurgery, London; ^d^Walton Centre, Liverpool	Cortese et al.^78^	30	–	–	X	–	–	–	X	Bianchi et al.,^79^ Cortese et al.^80^
23		University Hospital Nottingham, Nottingham	Chou et al.^81^	8	–	–	X	–	–	–	–	–
24		Nuffield Department of Clinical Neurosciences, Oxford	Matthews et al.^82^	18	17	X	–	–	–	X	–	Messina et al.^9^
25	UK,^e,f,g^ Italy^h^	^e^Nuffield Department of Clinical Neurosciences, Oxford; ^f^University of Cardiff, Cardiff; ^g^University of Nottingham, Nottingham; ^h^University of Siena, Siena	Matthews et al.^83^	44	–	–	X	–	–	–	–	Camera et al.,^84^ Messina et al.^9^
26	USA	MS Comprehensive Care Center, New York University, New York City	Lotan et al.^85^	47	37	X	X	–	–	–	–	–
27			Ventura et al.^86^	6/27	20	–	–	–	–	–	X	–

MUCCA, mean upper cervical cord area; T2 lesion, T2/FLAIR brain lesion volume.

The results of the meta-analyses are visualized using forest plots. Here, studies to the left of the vertical line (no effect) indicate lower mean volumes in the experimental group compared to healthy participants. Full forest plots with descriptive statistics are provided in the Supplemental Material (Figures S1–S5). Additionally, assessment of publication bias was visualized using funnel plots. Data points outside of the funnel, that is, further away from the pooled effect size (vertical line), point to lower precision of the effect size. A symmetrical funnel plot suggests no visual indication of publication bias or small-study effects.

### Whole brain volume

**Figure 2.**
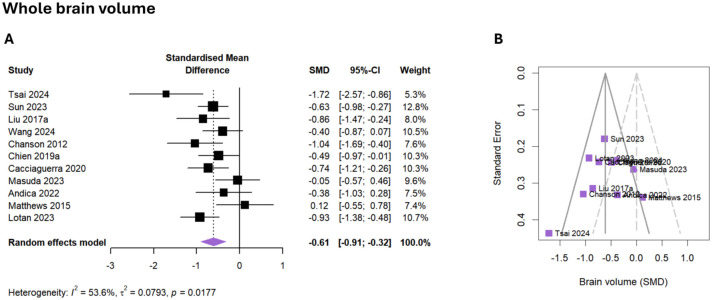
Meta-analysis of whole brain volume. (a) Forest plot showing the effect sizes of each study (squares) with their corresponding CIs (horizontal lines) and overall pooled effect size of the meta-analysis (diamond). (b) Funnel plot of whole brain volume SMD. CI, confidence interval; SMD, standardized mean difference.

The pooled effect size for brain volume was moderate and statistically significant (*g* = −0.61, 95% CI: −0.91 to −0.32, *p* < 0.001), revealing overall lower whole brain volumes in patients with AQP4-IgG+ NMOSD (Figure 2(a), Figure S1). Between-study heterogeneity was moderate (τ^2^ = 0.08, *I*^2^ = 53.6%). There were no associations with demographic or clinical variables, such as age, sex, or disease severity (EDSS).

A total of *k* = 11 studies (eligible: *k* = 21, removed due to multiple publications from the same cohort: *k* = 10) reporting 385 patients and 325 healthy participants were included in the meta-analysis of whole brain volume. There was no evidence of outliers or funnel plot asymmetry, indicating low risk of publication bias (intercept = −1.10, 95% CI: −5.05 to 2.85, *p* = 0.599, Figure 2(b)). Compared to a healthy control group, 5/11 (45%) studies found reduced whole brain volumes.^39,41,51,53,66^ In contrast, 6/11 studies (55%) report normal volumes in patients with AQP4-IgG+ NMOSD.^45,46,49,70,73,82^

### T2/FLAIR brain lesion volume

Sixteen studies were included in the analysis of T2/FLAIR brain lesion volumes (eligible: *k* = 38, multiple publication: *k* = 22), reporting 578 patients with AQP4-IgG+ NMOSD. A moderation analysis revealed a significant relationship between the average brain lesion volume and immunotherapy (β = 0.063, 95% CI: 0.018 to 0.108, *p* = 0.007). Specifically, the average brain lesion volume is expected to increase by 0.6 ml for each additional 10 immunotherapy treatments given in a sample. Here, the number of immunotherapy treatments was defined as the overall count of immunotherapeutic drugs administered to a sample, reflecting that some patients may have received several treatments in sequence.

No further association with demographic or clinical variables were observed. Lesions volumes were extracted from the entire brain in most studies (11/16). Five studies segmented white matter lesions only, referred to as “hyperintense white matter lesions.” Generally, most studies employed automated lesion segmentation tools (11/16). Manual lesion delineation was less common (5/16).

### Total gray matter volume

**Figure 3.**
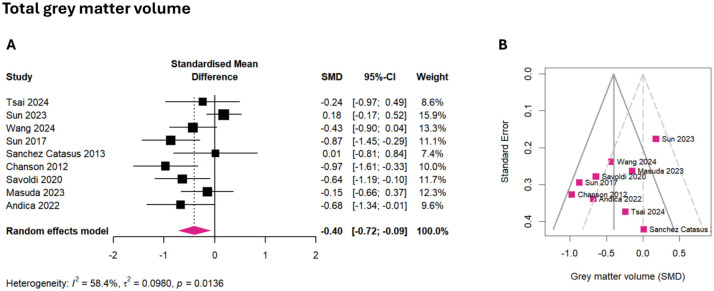
Meta-analysis of total gray matter volume. (a) Forest plot showing the effect sizes of each study (squares) with their corresponding CIs (horizontal lines) and overall pooled effect size of the meta-analysis (diamond). (b) Funnel plot of gray matter volume SMD. CI, confidence interval; SMD, standardized mean difference.

A small to moderate and statistically significant effect was seen in the meta-analysis of total gray matter volume (*g* = −0.40, 95% CI: −0.72 to −0.09, *p* = 0.018), which was lower in patients with AQP4-IgG+ NMOSD (Figure 3(a), Figure S2). Between-study heterogeneity was moderate (τ^2^ = 0.09, *I*^2^ = 58.4%). Brain lesion volume significantly predicted this effect size in a metaregression (β = 0.199, 95% CI: 0.049 to 0.349, *p* = 0.021), with the effect size estimate expected to rise by 0.2 for every additional milliliter in brain lesion volume.

The meta-analysis of total gray matter volume included *k* = 9 studies (eligible: *k* = 17, multiple publication: *k* = 8), representing 259 patients and 267 healthy participants. No outliers were detected. Egger’s regression showed no funnel plot asymmetry, suggesting low risk of publication bias (intercept = −3.21, 95% CI: −6.73 to 0.32, *p* = 0.118, Figure 3(b)). Four out of the eight (50%) studies with group comparisons observed reduced gray matter volume compared to healthy participants,^41,47,62,70^ while another 4/8 (50%) found no group difference.^46,49,51,73^

### Total white matter volume

No statistically significant effect was observed for the pooled effect size of total white matter volume (*g*_adj_ = −0.09, 95% CI: −0.80 to 0.99, *p* = 0.822, Figure S3(A)–(C)), and between-study heterogeneity was substantial (τ^2^ = 1.36, *I*^2^ = 89.2%). In a metaregression, sex emerged as a significant predictor (β = 0.046, 95% CI: 0.002 to 0.091, *p* = 0.045), with the effect size expected to rise by 0.05 for every additional percentage point of female patients in the sample.

Initially, *k* = 7 studies (eligible: *k* = 14, multiple publication: *k* = 7) with data from 269 patients and 299 healthy participants were included. One study was identified as an outlier (Tsai et al., 2024), resulting in funnel plot asymmetry (Egger’s regression: intercept = −5.74, 95% CI: −9.90 to −1.57, *p* = 0.043). To correct for a potential publication bias, we adjusted the pooled effect size estimate using Duval and Tweedie’s trim and fill method. Following adjustment, Egger’s regression test was unremarkable (intercept = −0.73, 95% CI: −7.03 to −5.57, *p* = 0.827, Figure S3(B)). Compared to matched healthy participants, 3/7 (43%) studies reported reduced white matter volume,^39,46,51^ while 4/7 (57%) reported normal volumes in patients with AQP4-IgG+ NMOSD.^41,49,62,70^

### Thalamus volume

The pooled effect size for thalamus volume was small, but not significant (*g* = −0.20, 95% CI: −0.48 to −0.07, *p* = 0.116, Figure S4(A)), with low heterogeneity (τ^2^ = 0.0, *I*^2^ = 0.0%) and a tendency toward lower thalamic volumes in patients with AQP4-IgG+ NMOSD. Age significantly predicted the effect size in an metaregression (β = 0.044, 95% CI: 0.002 to 0.085, *p* = 0.045).

Included were *k* = 7 studies (eligible: *k* = 10, multiple publication: *k* = 3), collectively involving 153 patients and 163 healthy participants. No outliers were identified, and Egger’s regression did not detect asymmetry in the funnel plot, indicating minimal risk of publication bias (intercept = −1.33, 95% CI: −6.59 to 3.94, *p* = 0.643, Figure S4(B)). Most studies (6/7, 86%) reported normal thalamic volumes compared to healthy controls.^39,45,50,60,73,82^ One study reported reduced thalamic volume (1/7, 14%).^62^

### Mean upper cervical cord area

**Figure 4.**
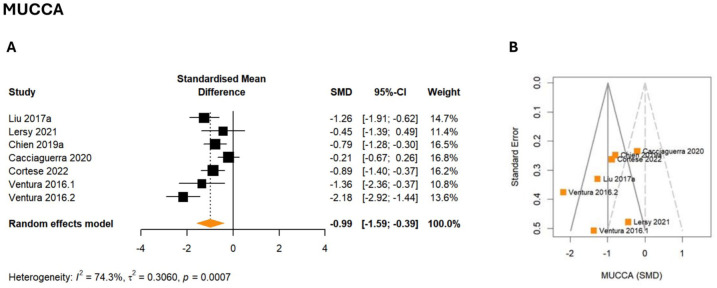
Meta-analysis of the MUCCA. (a) Forest plot showing the effect sizes of each study (squares) with their corresponding CIs (horizontal lines) and overall pooled effect size of the meta-analysis (diamond). (b) Funnel plot of MUCCA SMD. CI, confidence interval; MUCCA, mean upper cervical cord area; SMD, standardized mean difference.

The meta-analysis revealed a large and statistically significant pooled effect size for the MUCCA (*g* = −0.99, 95% CI: −1.59 to −0.39, *p* = 0.007), which was lower in patients with AQP4-IgG+ NMOSD (Figure 4(a), Figure S5). Between-study heterogeneity was moderate to substantial (τ^2^ = 0.31, *I*^2^ = 74.3%). MUCCA effect sizes were not associated with demographic or clinical variables in the metaregression.

The meta-analysis incorporated *k* = 7 studies (eligible: *k* = 11, multiple publication: *k* = 4), which together reported on 189 patients and 162 healthy participants. No potential outliers were detected. Egger’s test did not show significant funnel plot asymmetry, suggesting that publication bias is unlikely (intercept = −3.48, 95% CI: −8.66 to 1.70, *p* = 0.245, Figure 4(b)). Almost all studies (6/7, 86%) observed a reduced MUCCA,^45,52,53,66,78,86^ while one study found no difference compared to healthy control participants.^66^

### Bias analysis

None of the MRI outcomes showed an association with year of publication in a meta-regression, suggesting that potential technical improvements in automated segmentation over time did not impact the conclusions. Neither mean volumes nor effect sizes from the meta-regression were associated with differences in data acquisition or segmentation approaches between the studies (see Supplemental Table 1).

### Voxel-based morphometry

We identified 25 studies reporting whole-brain gray matter volumetry in patients with NMOSD. Fifteen studies were excluded due to samples with mixed or undocumented serostatus. The remaining 10 studies reported samples with exclusively AQP4-seropositive patients. Out of these, three studies were removed after screening for multiple reports.^15,87,88^ This resulted in a final selection of seven unique studies, reporting data from *N* = 233 patients and *N* = 207 healthy participants from seven clinical centers in China,^89–91^ India,^92^ Italy,^62^ Japan,^93^ and the United Kingdom.^82^

**Figure 5.**
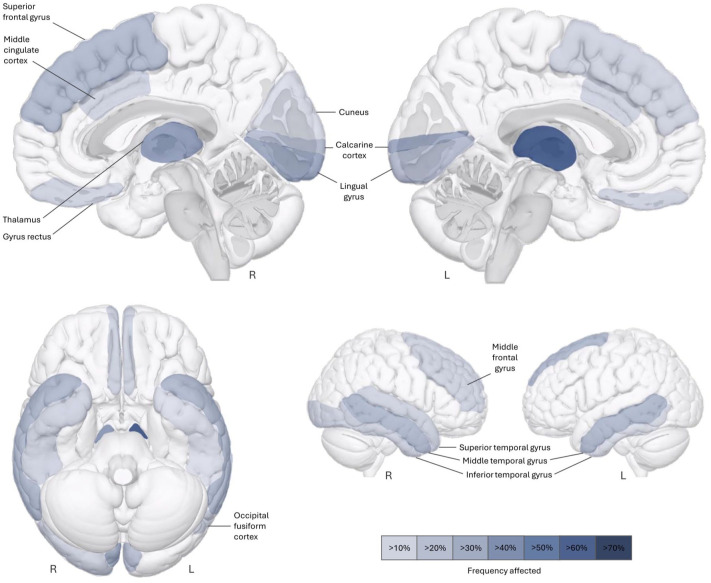
Prevalence of gray matter volume reductions in patients with AQP4-IgG+ NMOSD using whole-brain VBM analysis. Across a total of 233 patients and 7 nonoverlapping study samples, reduced volumetric estimates were most frequently observed in the thalamus and occipital cortex, but also extended to frontal and temporal cortical regions. AQP4-IgG+ NMOSD, aquaporin-4-IgG seropositive neuromyelitis optica spectrum disorder; VBM, voxel-based morphometry.

Significant reductions in gray matter volumetric estimates were most frequently observed in the thalamus (*left*: 69%/*right*: 40%) and the occipital lobe (44%), including the bilateral lingual gyrus (25%/34%), bilateral calcarine cortex (34%/25%), left occipital fusiform cortex (16%), right inferior occipital gyrus (16%), and right cuneus (11%, Figure 5). Less frequently, cortical gray matter reductions were observed in the frontal lobe, especially in the superior frontal gyrus (27%/16%), and the temporal cortex, particularly the bilateral middle temporal gyrus (27%/27%). One of the seven studies reported no difference compared to healthy controls,^82^ and none reported an increase in gray matter volumetric estimates.

## Discussion

**Figure 6.**
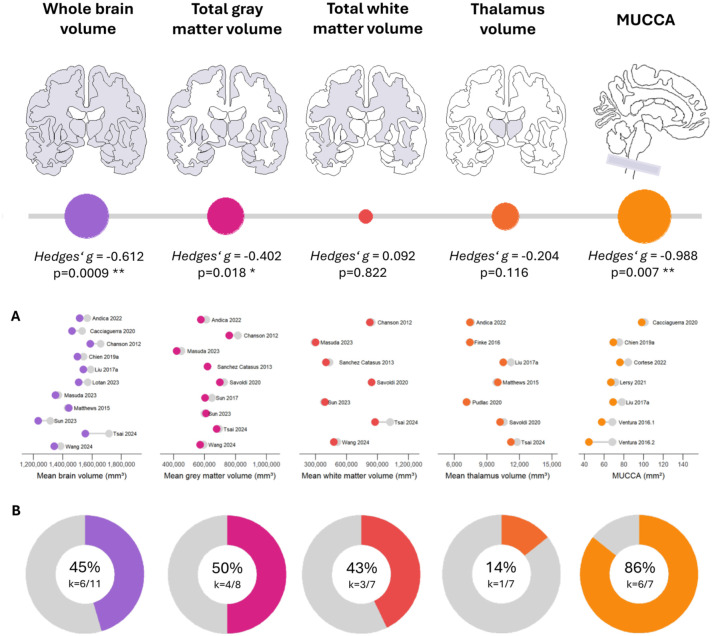
Brain volumetry and spinal cord MRI in AQP4-IgG+ NMOSD (meta-analysis). Pooled effect sizes, (a) study means relative to the corresponding control group of age- and sex-matched healthy participants (gray), and (b) the proportion of studies reporting statistically significant reductions in volume/area compared to control participants. AQP4-IgG+ NMOSD, aquaporin-4-IgG seropositive neuromyelitis optica spectrum disorder; MRI, magnetic resonance imaging.

Our meta-analysis of MRI studies in AQP4-IgG+ NMOSD revealed moderate-to-large effects for whole brain volume (*g* = −0.61, *p* < 0.001), gray matter volume (*g* = −0.40, *p* = 0.018), and the MUCCA (*g* = −0.99, *p* = 0.007), which were all reduced in patients compared to matched healthy controls. In contrast, white matter volume (*g*_adj_ = −0.09) and thalamus volume (*g* = −0.20) showed no or only small effects, with varying degree of heterogeneity between studies (Figure 6). Potential biases, if present, were accounted for during study inclusion and statistical analyses, minimizing the possibility that small study effects, year of publication, and sample duplication affected these findings. In a systematic review of voxelwise assessments of cortical and subcortical estimated volume loss (VBM), we found that reductions are most frequently observed in the bilateral thalamus, occipital cortex, and temporal and frontal cortical areas. Taken together, these findings suggest that reduced volumes can be found both globally (i.e., on the whole brain level) and locally (i.e., on the level of specific brain areas) in patients with AQP4-IgG+ NMOSD.

The moderate effect for whole brain volume decrease found in this meta-analysis may be driven by gray matter changes to a certain extent, given that we also observed a significant effect for total gray matter volume, but no effect for white matter volume decrease. Total white matter volumes in patients with AQP4-IgG+ NMOSD also showed high heterogeneity across studies. Additionally, our metaregression revealed that total gray matter volumes were related to brain lesion volumes reported in the included studies. A recent retrospective multicenter study observed no correlation between brain lesion volume and gray matter volume in patients with AQP4-IgG+ NMOSD (*N* = 135), which was different to the patterns seen in the MS and MOGAD samples.^15^ Occipital gray matter volume was reduced when compared to non-age matched healthy participants in this study. Interestingly, when only patients with AQP4-IgG+ NMOSD without lesions were considered, this difference disappeared. These findings indicate that the influence of brain lesions on gray matter integrity in AQP4-IgG+ NMOSD may be more intricate. Further research is necessary to determine potential nonlinear longitudinal trajectories of the disease on gray matter integrity. In addition, future longitudinal volumetric imaging studies may investigate volumetric changes outside of clinical attacks. This may provide insights into potential paths to recovery or evidence for relapse-independent disease activity.^94,95^

In fact, a large international study found that brain lesions are more common in AQP4-IgG+ NMOSD than previously thought, although typical spinal cord longitudinally extensive transverse myelitis and optic nerve lesions were also very prevalent.^10^ Interestingly, the most predominant brain imaging finding were non-specific white matter lesions, suggestive of small-vessel disease in ~20% of the patients. Aging, cardiovascular risk factors, and associated comorbidities may thus weave into the neuropathological profile of AQP4-IgG+ NMOSD, especially as they increase the likelihood of T2/FLAIR lesion accrual and patients are often diagnosed at an older age.^96,97^ For instance, our metaregression observed an effect of age for thalamus volume, where the effect size is expected to rise by 0.04 for every additional year of age. Disease-characteristic brain lesions like T1 black holes, juxtacortical lesions, or Dawson fingers seen in MS are usually absent in AQP4-IgG+ NMOSD. Instead, quantitative probability mapping showed that lesions were less frequent, smaller, and had a more variable distribution throughout the brain, much like typical nonspecific white matter lesions.^83^ It is uncommon that “disease-specific” brain lesions are segmented for volumetric analysis in AQP4-IgG+ NMOSD, where most lesions may be referred to as “T2-hyperintensities” or “T2-weighted lesions,” which are mostly non-disease specific in nature. Brain lesion subsegmentations with more clearly described regions (i.e., periventricular or pons lesions)^98^ for volumetric extraction may aid research in lesion profiles in the future.

Our meta-analysis revealed a large effect for the reduced MUCCA observed in all included studies. In contrast to brain lesions, inflammatory spinal cord lesions in the context of myelitis attacks occur in a large majority of patients with AQP4-IgG+ NMOSD. They can equally affect the cervical and thoracic cord, and around two-third of the patients have been observed to retain chronic spinal cord lesions.^57^ Driven mainly by axonal demyelination, they play a significant role in long-lasting spinal cord-related disability.^99^ However, spinal cord lesions are rarely segmented by volume since this requires a dedicated spinal cord MRI sequence and fully automated tools are not available. MUCCA has been suggested as an alternative measure for spinal cord atrophy. In a comparative study, MUCCA showed a similar discriminatory performance between patients and healthy participants as total spinal or cervical cord volume, even though MUCCA can be measured from cerebral MRI scans covering the upper cervical cord.^56^ Interestingly, lower MUCCA that was related to general disability was also found in patients without previous myelitis or visible spinal cord lesions.^86^ There is now evidence that lesions may precede a clinical attack, but not long enough to be considered a silent lesion.^100^ Thus, it would seem that spinal cord lesions and atrophy may occur close to an attack, but not necessarily in an acute manner. When looking at the gray matter versus white matter in the upper cervical cord, there have been findings that patients with a myelitis attack show increased gray matter percentage, possibly indicating astrogliosis in AQP4-IgG+ NMOSD.^55^ This would, of course, complicate findings in MUCCA, which does not distinguish between gray and white matter in the cervical cord; therefore, further investigation of spinal cord lesion and atrophy dynamics in this disease is warranted.

Our systematic review of VBM revealed that volume reductions in thalamic areas were among the most common deep gray matter findings, affecting both the left (69%) and the right thalamus (40%). In contrast, our meta-analysis of thalamus volume showed a small effect that did not reach statistical significance. Six out of seven included studies did not find a statistical group difference in comparison to healthy control participants,^39,45,50,60,73,82^ only one study reported lower thalamic volumes in patients with AQP4-IgG+ NMOSD.^62^ It stands to reason that these findings can be attributed to differences in volumetric extraction methods, or possibly due to cohort differences. In the case of VBM,^101,102^ gray matter volume estimates in both cortical and subcortical areas are compared between groups on a voxel-by-voxel manner. The smallest detectable difference therefore equals the size of a single voxel in the input T1 scan. This differs from subcortical segmentation algorithms, as implemented in FSL FIRST^19^ or FreeSurfer,^103^ that create participant-level parcellations of specific brain structures from which volumes can be extracted. Extracting a volume estimate from the entire thalamic structure may therefore miss more subtle or localized volume reductions. On the other hand, VBM may be more prone to partial volume effects, where a single voxel contains a mixture of the tissue types the segmentation was supposed to distinguish between (e.g., gray matter and cerebrospinal fluid). The chance of these errors is higher for large voxel resolutions and can introduce the risk of false positives. Therefore, subfield/subnuclei volumetric investigations may offer a more fine-grained view of thalamic integrity. For instance, patients with AQP4-IgG+ NMOSD and optic neuritis had lower volumes of the lateral geniculate nucleus, a major thalamic component of the visual pathway.^61,104^ In another study, volumes of the ventral posterior nucleus, a thalamic nucleus involved in relaying sensory information, were associated with the intensity of neuropathic pain in patients with AQP4-IgG+ NMOSD.^54^

We observed no effect for total white matter volume in this meta-analysis. Other forms of white matter damage, such as reduced neurite density reflecting axonal loss in tracts with white matter lesions^75,105^ or secondary demyelination of normal-appearing white matter,^106^ can be observed using diffusion and myelin water imaging. Sex emerged as a significant predictor of total white matter volume in our meta-analysis, with lower white matter volume associated with a greater proportion of female patients in the sample. AQP4-IgG+ NMOSD has a high prevalence in women (about 9:1).^107,108^ This predominance still increases during fertile age, where progesterone increases the risk of developing AQP4-IgG+ NMOSD^109^ and relapses were observed to become more frequent during the time around pregnancy and postpartum when immunosuppressive treatments might be discontinued.^110,111^ This is also reflected in overall higher annualized relapse rates in women with AQP4-IgG+ NMOSD.^112^ Notably, younger women (⩽40 years) were also found to be more likely to respond to treatment and show remission than older women.^107^ While we did not observe an effect of sex on whole brain volume, about 90% of patients with relapsing NMOSD are women. Including sex as a disease-related variable may thus be important for treatment decisions.^113^ On the whole, additional studies are needed to explore how brain imaging features differ between women and men with AQP4-IgG+ NMOSD and which hormonal and environmental factors impact neuroinflammatory and neurodegenerative processes.

Our study has several limitations. First, the rarity of AQP4-IgG+ NMOSD poses distinct challenges. Several studies had to be excluded due to a lack of demographic matching between patients with AQP4-IgG+ NMOSD and the healthy participant group. Cohorts of patients with NMOSD are at times reported and compared with a group of MS patients. If present, the healthy control group may be matched to the MS group and are thus often significantly younger than the patients with AQP4-IgG+ NMOSD. To avoid a potential bias, these studies were excluded, resulting in a lower number of available studies. At the same time, the reporting of clinical attacks varies between studies and cognitive impairment is assessed using heterogeneous protocols. Standardizing the reporting and harmonizing neuropsychological assessments may offer new insights in future meta-analyses—such as the effect of optic neuritis attack on regional brain volume.

Second, this review accounted for several major biases, including small study effects, citation and publication biases, technological developments over time, as well as multiple publications that are based on overlapping patient cohorts. However, we cannot fully exclude an outcome reporting or diagnostic bias.^29^ If multiple MRI analyses are conducted for a study, some researchers may be inclined to drop analyses with null or unfavorable results, which then remain unreported. There has been some speculation that inclusion in studies is based on clinical attack characteristics as well, which would bias findings toward associations with myelitis and/or optic neuritis. While this is a general problem of meta-analyses, we countered this bias by including all eligible volumetric data regardless of the direction of the effect. With regard to the detection of AQP4 antibodies, cell-based assays have been shown to be most sensitive and specific.^114^ Thus, future imaging studies in patients with NMOSD would benefit from consistent detailing of the diagnostic assay used for diagnosis.

The extraction of lesion volumes showed some degree of variability. Most included studies segmented lesions from the entire brain tissue. A few studies extracted lesions from the white matter only, yet we believe that these measures are correlated. With regard to lesion segmentation methods, previous research in MS has shown that automated and manual lesion tracing yields robust and comparable results.^115–117^ Albeit of relevant for infratentorial relapses, studies reporting volumetric estimates of the cerebellum and brainstem are still scarce. Lastly, this meta-analysis was limited to English-language publications. Eligible studies were identified without geographic restriction, and data from 16 countries were included. Nevertheless, global disparities in access to, and affordability of MRI,^118,119^ together with inconsistent or absent reporting of ethnicity across studies, did not allow for identification of potential risk related to geographic location or ethnic background within the scope of this meta-analysis. Several previous studies have endeavored to thoroughly describe these ethnic differences.^120–122^

## Conclusion

In conclusion, our meta-analysis revealed that reduced whole brain and gray matter volumes, together with a lower cross-sectional area of the upper cervical cord (MUCCA), are characteristic MRI markers of AQP4-IgG+ NMOSD. Moreover, findings of reduced gray matter volume in the thalamus, occipital, frontal, and temporal cortices provide additional evidence for a more widespread CNS involvement in this neuroimmunological disease. This research has important implications for the choice of radiological endpoints in clinical trials, monitoring patients, and may inform future MRI-based research seeking to evaluate disease progression in NMOSD.

## Supplemental Material

sj-docx-1-tan-10.1177_17562864251394843 – Supplemental material for Brain volumetry and spinal cord imaging in patients with AQP4-IgG+ NMOSD—a systematic review and meta-analysis Supplemental material, sj-docx-1-tan-10.1177_17562864251394843 for Brain volumetry and spinal cord imaging in patients with AQP4-IgG+ NMOSD—a systematic review and meta-analysis by Josephine Heine and Claudia Chien in Therapeutic Advances in Neurological Disorders

sj-docx-2-tan-10.1177_17562864251394843 – Supplemental material for Brain volumetry and spinal cord imaging in patients with AQP4-IgG+ NMOSD—a systematic review and meta-analysis Supplemental material, sj-docx-2-tan-10.1177_17562864251394843 for Brain volumetry and spinal cord imaging in patients with AQP4-IgG+ NMOSD—a systematic review and meta-analysis by Josephine Heine and Claudia Chien in Therapeutic Advances in Neurological Disorders

## Declarations

**Ethics approval and consent to participate:** Not applicable (data obtained from published records).

**Consent for publication:** Not applicable (data obtained from published records).

**Availability of data and materials:** The datasets generated and analyzed during the current study are available from the corresponding author upon request.
