# Supplementary material for: Brain volumetry and spinal cord imaging in patients with AQP4-IgG+ NMOSD—a systematic review and meta-analysis
Source: Ther Adv Neurol Disord. 2025 Dec 6;18:17562864251394843. doi: 10.1177/17562864251394843 (PMC12681629; doi:10.1177/17562864251394843)
Supplement: sj-docx-2-tan-10.1177_17562864251394843 – Supplemental material for Brain volumetry and spinal cord imaging in patients with AQP4-IgG+ NMOSD—a systematic review and meta-analysis [file sj-docx-2-tan-10.1177_17562864251394843.docx]

**Supplementary material**

**Supplementary Methods**

**List of key journals searched to identify eligible studies**

American Journal of Neuroradiology; Annals of Clinical and Translational Neurology; Annals of Neurology; Archives of Neurology; Biological Psychiatry; BMC Neurology; BMJ Neurology Open; Brain; Brain Communications; Clinical Neurology and Neurosurgery; Clinical Neurophysiology; Cortex; European Journal of Neurology; European Neurology; Frontiers in Immunology, Frontiers in Neurology; JAMA; JAMA Neurology; Journal of Cerebral Blood Flow & Metabolism; Journal of Clinical Neurology; Journal of Clinical Neurophysiology; Journal of Clinical Neuroscience; Journal of Neuroimaging; Journal of Neuroimmunology; Journal of Neuroinflammation; Journal of Neurology; Journal of Neuroradiology; Journal of the Neurological Sciences; Multiple Sclerosis Journal - Experimental, Translational and Clinical; Multiple Sclerosis and Related Disorders; Multiple Sclerosis Journal; Nature Neuroscience; Nature Reviews Neurology; Neurodegenerative Diseases; NeuroImage; NeuroImage: Clinical; Neurological Research; Neurology; Neurology and Neuroscience; Neurology: Clinical Practice; Neurology Clinics; Neurology Neuroimmunology & Neuroinflammation; Neurology, Psychiatry and Brain Research; Neurorehabilitation & Neural Repair; Neurosurgery & Psychiatry; PLOS one; Practical Neurology; Radiology; Science; Scientific Reports, The Journal of Neuropsychiatry and Clinical Neurosciences; The Lancet, The Lancet Neurology

**Supplementary Table 1**

**Table S1. Effect of methods.** The non-parametric Kruskal-Wallis test did not show any influence of scanner model, field strength, voxel size, lesion filling, and segmentation method on any of the investigated volumes. Abbreviations: MUCCA – mean upper cervical cord area.

| **Volume** |  | **Type of scanner** | **Field strength** | **Voxel size** | **Lesion filling** | **Segmentation method** |
| --- | --- | --- | --- | --- | --- | --- |
| **Whole brain** | *Mean volume* | H(2)=0.00, p=1.00 | H(1)=0.83, p=0.36 | H(2)=4.50, p=0.11 | H(1)=0.00, p=1.00 | H(3)=5.80, p=0.12 |
|  | *Hedges’ g* | H(2)=3.98, p=0.13 | H(1)=1.20, p=0.27 | H(2)=4.82, p=0.09 | H(1)=0.96, p=0.33 | H(3)=3.37, p=0.34 |
| **T2/FLAIR brain lesion** | *Mean volume* | H(2)=0.15, p=0.93 | H(2)=1.36, p=0.51 | H(2)=1.23, p=0.54 | - | H(3)=1.11, p=0.77 |
|  | *Hedges’ g* | - | - | - | - | - |
| **Total grey matter** | *Mean volume* | H(2)=4.11, p=0.13 | H(1)=0.07, p=0.80 | H(2)=3.57, p=0.17 | H(1)=0.02, p=0.88 | H(4)=6.87, p=0.14 |
|  | *Hedges’ g* | H(2)=1.81, p=0.41 | H(1)=0.07, p=0.80 | H(2)=2.14, p=0.34 | H(1)=0.02, p=0.88 | H(4)=2.84, p=0.58 |
| **Total white matter** | *Mean volume* | H(2)=2.38, p=0.30 | H(1)=1.13, p=0.29 | H(2)=2.70, p=0.26 | H(1)=0.13, p=0.72 | H(3)=5.14, p=0.16 |
|  | *Hedges’ g* | H(2)=1.24, p=0.54 | H(1)=0.13, p=0.72 | H(2)=1.80, p=0.41 | H(1)=0.13, p=0.72 | H(3)=1.46, p=0.69 |
| **Thalamus** | *Mean volume* | H(2)=0.29, p=0.87 | H(1)=0.15, p=0.70 | H(2)=3.57, p=0.17 | H(1)=1.19, p=0.28 | H(1)=1.93, p=0.17 |
|  | *Hedges’ g* | H(2)=1.14, p=0.57 | H(1)=1.35, p=0.25 | H(2)=3.57, p=0.17 | H(1)=0.05, p=0.83 | H(1)=0.86, p=0.35 |
| **MUCCA** | *Mean volume* | H(1)=3.00, p=0.08 | H(1)=1.35, p=0.25 | H(1)=0.20, p=0.66 | - | H(3)=3.57, p=0.31 |
|  | *Hedges’ g* | H(1)=0.33, p=0.56 | H(1)=0.15, p=0.70 | H(1)=1.80, p=0.18 | - | H(3)=4.71, p=0.19 |

**Supplementary Figures**

**
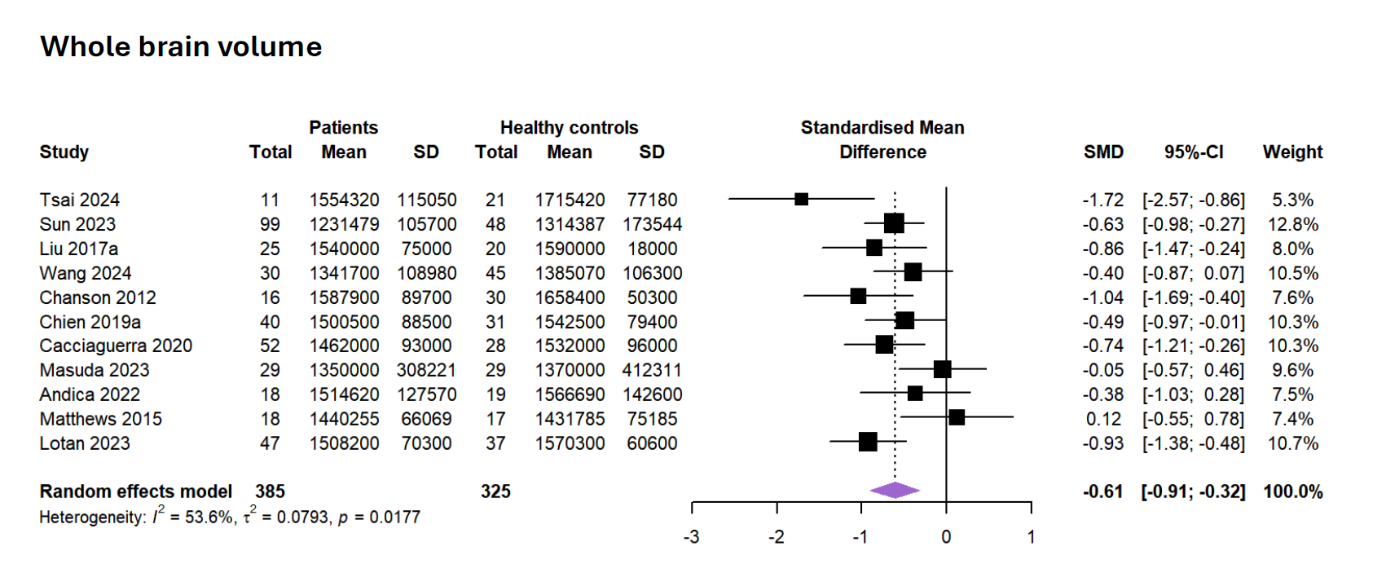
**

**Fig S1. Extended forest plot for meta-analysis of whole brain volume.** Forest plot showing the effect sizes of each study (squares) with their corresponding confidence intervals (horizontal lines) and overall pooled effect size of the meta-analysis (diamond). Abbreviations: CI – confidence interval, SD – standard deviation, SMD – standardised mean difference.

**
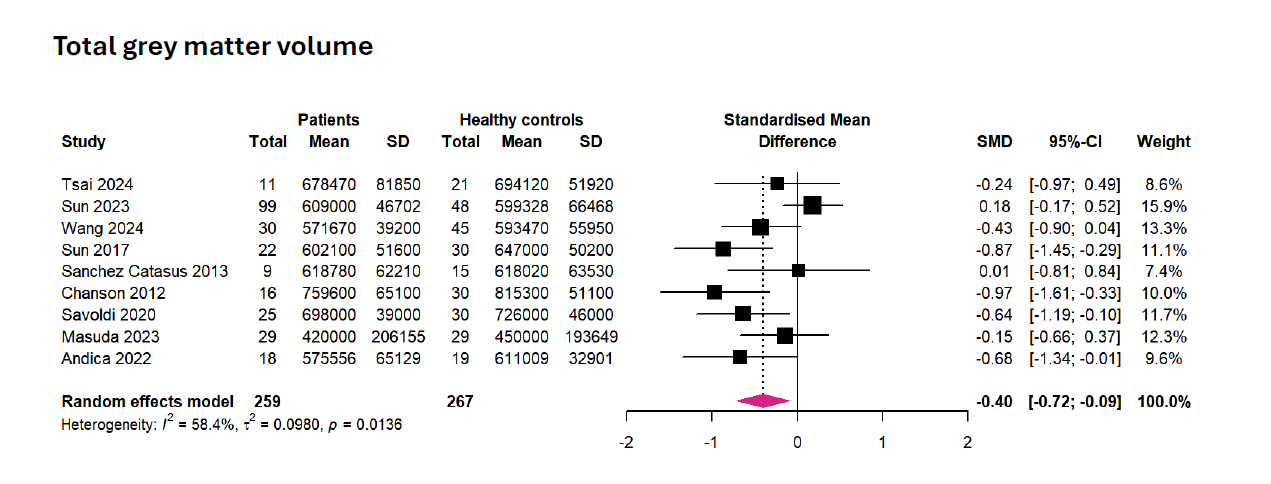
**

**Fig S2. Extended forest plot for meta-analysis of total grey matter volume.** Forest plot showing the effect sizes of each study (squares) with their corresponding confidence intervals (horizontal lines) and overall pooled effect size of the meta-analysis (diamond). Abbreviations: CI – confidence interval, SD – standard deviation, SMD – standardised mean difference.

**
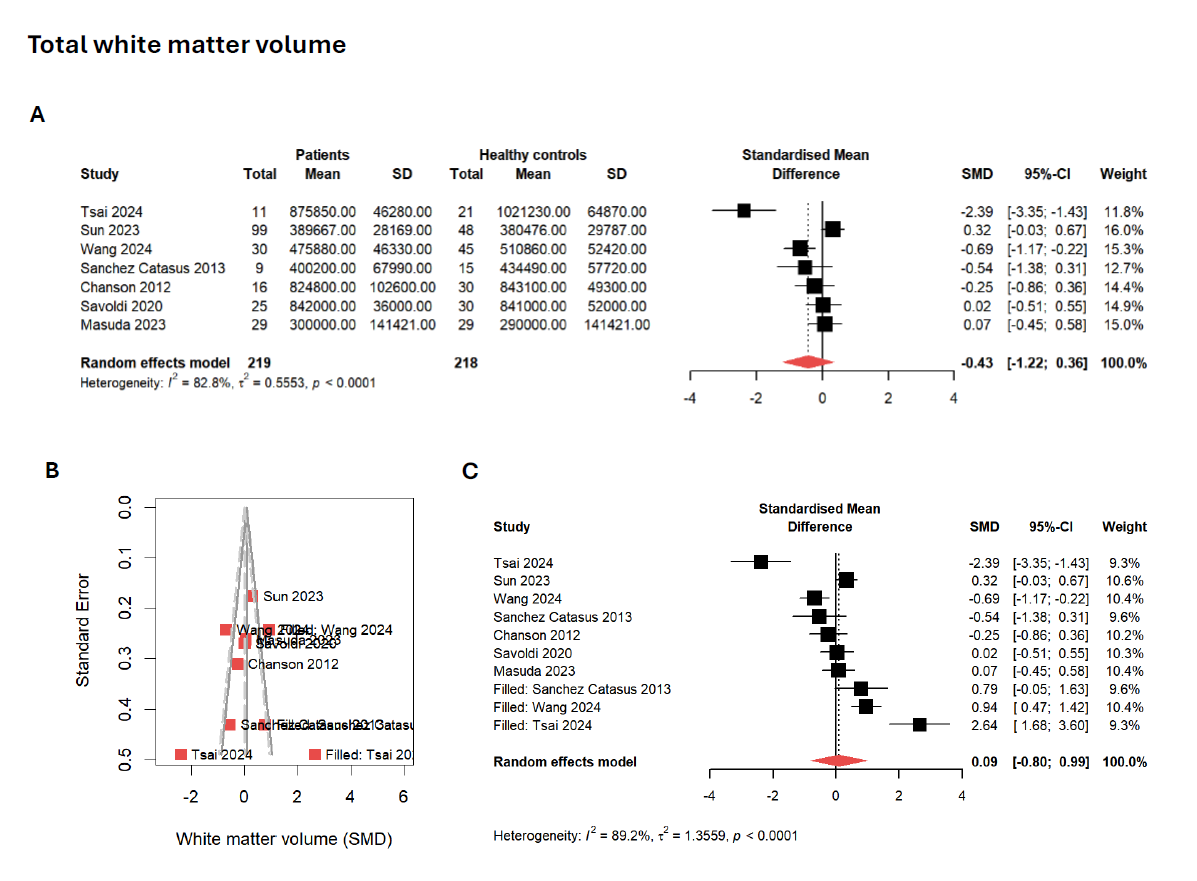
**

**Fig S3. Extended forest plot for meta-analysis of total white matter volume.** **(A)** Forest plot before correction for small study effects. **(B)** Funnel plot of whole brain white matter volume SMD after correction for small-study effects. **(C)** Forest plot after correction using the Duval and Tweedie trim and fill method. Abbreviations: CI – confidence interval, SD – standard deviation, SMD – standardised mean difference.

**
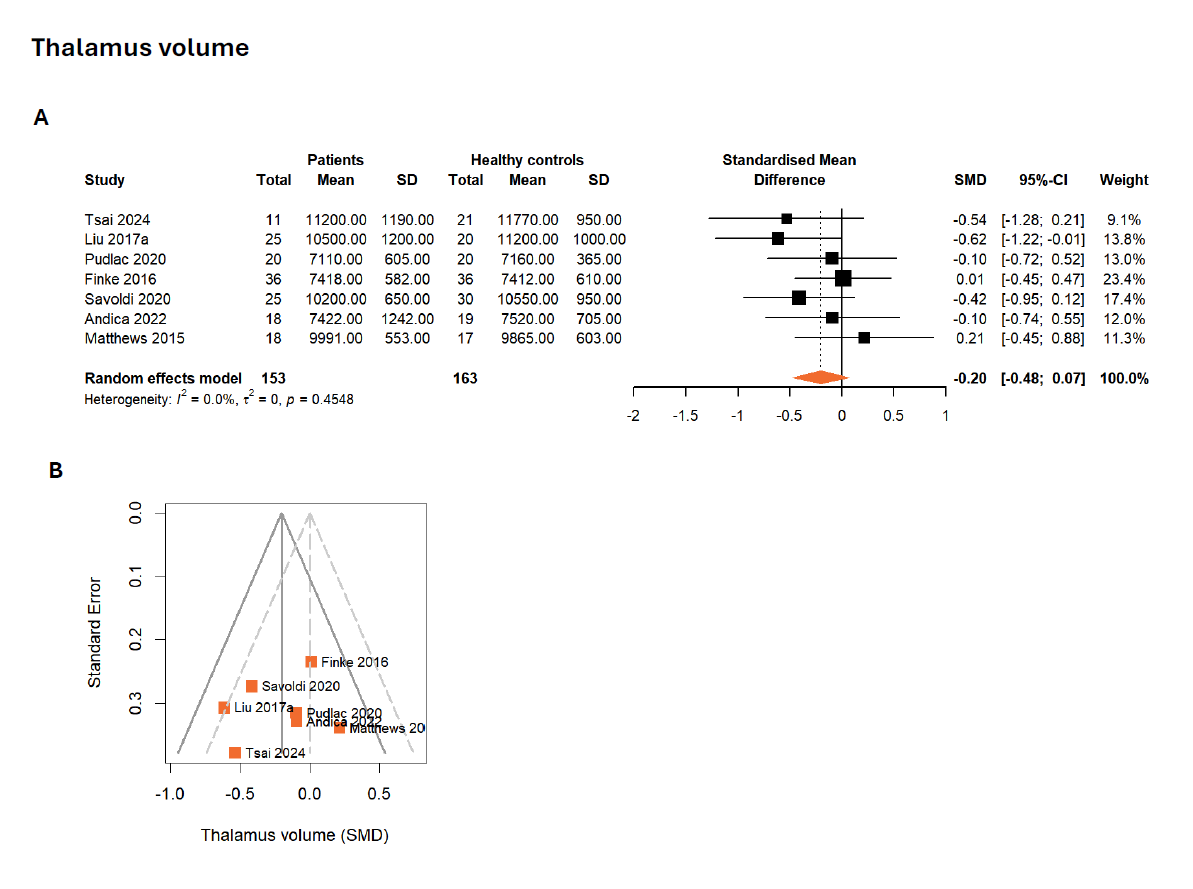
**

**Fig S4. Extended forest plot for meta-analysis of thalamus volume.** **(A)** Forest plot showing the effect sizes of each study (squares) with their corresponding confidence intervals (horizontal lines) and overall pooled effect size of the meta-analysis (diamond). **(B)** Funnel plot of thalamus SMD. Abbreviations: CI – confidence interval, SD – standard deviation, SMD – standardised mean difference.

**
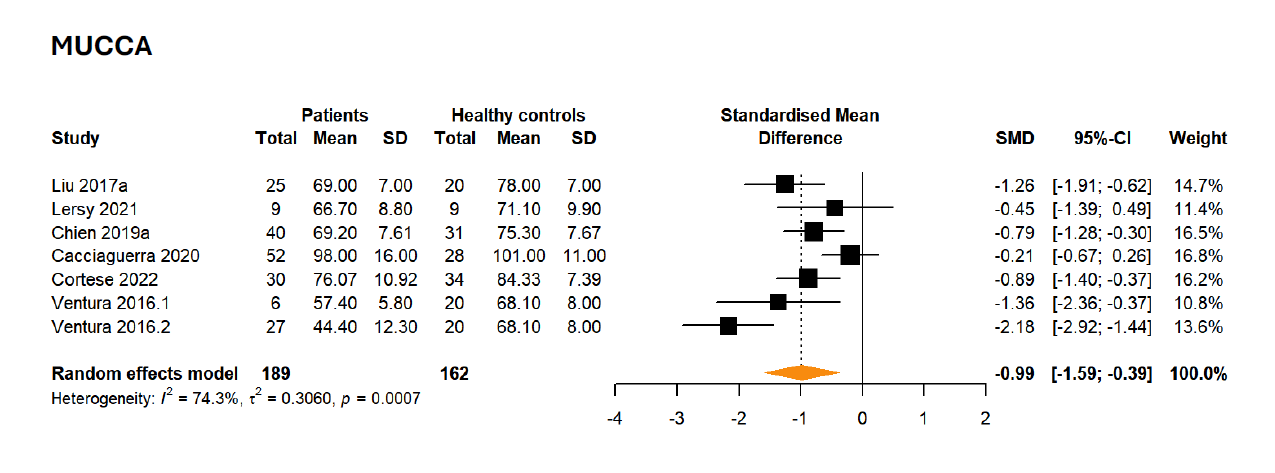
**

**Fig S5. Extended forest plot for meta-analysis of the mean upper cervical cord area (MUCCA).** Forest plot showing the effect sizes of each study (squares) with their corresponding confidence intervals (horizontal lines) and overall pooled effect size of the meta-analysis (diamond). Abbreviations: CI – confidence interval, SD – standard deviation, SMD – standardised mean difference.
